# Unilateral Exoskeleton Training for Balance/Gait in Hemiplegic Stroke: Pilot Study

**DOI:** 10.1155/bn/4208995

**Published:** 2026-09-27

**Authors:** Shaoting Zhou, Sen Wang, Xueying Qu, Jing Fan, Hanlin Xu, Linyu Chen, Lingyu Liu

**Affiliations:** ^1^ Department of Neurology and Neurological Rehabilitation, Shanghai Disabled Persons′ Federation Key Laboratory of Intelligent Rehabilitation Assistive Devices and Technologies, Shanghai Yangzhi Rehabilitation Hospital (Shanghai Sunshine Rehabilitation Center), Tongji University School of Medicine, Shanghai, China, tongji.edu.cn; ^2^ Department of Orthopedic Rehabilitation, Shanghai Yangzhi Rehabilitation Hospital (Shanghai Sunshine Rehabilitation Center), Tongji University School of Medicine, Shanghai, China, tongji.edu.cn

**Keywords:** rehabilitation, stroke, unilateral-lower limb exoskeleton rehabilitation training robot, walking function

## Abstract

**Objective:**

The aim of this study is to evaluate the effects of a unilateral‐lower limb exoskeleton rehabilitation training robot on balance and lower limb function after stroke.

**Methods:**

In this pilot study, stroke patients with hemiplegic at Tongji University Yangzhi Rehabilitation Hospital (between October 2022 and December 2023) were randomly assigned to either the robot group or the control group. The primary measure was the Berg balance scale (BBS), whereas secondary measures included the 10‐m walking time test, 6‐min walk test, three‐dimensional gait analysis, and pain levels using the Hospital Anxiety and Depression Scale (HADS) and visual analogue scale (VAS).

**Results:**

Among 36 post‐stroke hemiplegia patients (25 males; 18 per group), the robot group significantly outperformed the traditional group. The robot group improved BBS scores (5.50 ± 2.09 vs. 3.11 ± 1.50, p < 0.001), 6‐min walking distance (15.89 ± 8.64 m vs. 9.73 ± 5.83 m, p = 0.017), and 10‐m walking time reduction (8.82 ± 3.22 s vs. 6.83 ± 1.81 s, p = 0.030). Gait parameters also showed greater gains: hip flexion (3.57°±2.30° vs. 1.72°±2.15°, p = 0.018), knee flexion (5.28°±4.89° vs. 2.63°±2.37°, p = 0.046), and ankle dorsiflexion (4.81°±5.57° vs. 1.09°±4.19°, *p* = 0.030). Depression scores improved more in the robot group (2.89 ± 1.75 vs. 1.61 ± 1.20, p = 0.015). No significant differences occurred in anxiety or pain VAS scores (p > 0.05).

**Conclusion:**

Integrating a unilateral‐lower limb exoskeleton rehabilitation robot with traditional therapy significantly enhances lower limb recovery in stroke patients with hemiplegia. Future large‐scale randomized controlled trials and follow‐up evaluations are needed to validate the current findings.

## 1. Introduction

Stroke is a prevalent neurological condition characterized by a sudden onset of neurologic dysfunction attributable to focal brain ischemia [1] or brain hemorrhage [2, 3]. Approximately 25% of adults will experience a stroke during their lifetime, with about 70% of these events classified as ischemic strokes [4]. Stroke is associated with a high rate of disability, with up to 80% of affected individuals suffering from residual motor dysfunction post‐stroke [5]. Notably, lower limb motor impairments are particularly pronounced [6]. Such impairments often manifest as muscle weakness, inadequate weight transfer, restricted mobility, balance disorders, and gait dysfunction [7, 8], significantly compromising patients′ abilities to perform daily activities and diminishing their overall quality of life. Consequently, both stroke and the resultant disabilities impose a substantial burden on patients, their families, and society at large. Although some patients may experience spontaneous functional recovery after a stroke, timely and effective rehabilitation training remains indispensable for the majority [9]. The restoration of lower limb motor function is a critical component of the rehabilitation process for individuals with hemiplegic stroke [10, 11]. Early interventions emphasizing balance and gait training have been shown to yield positive outcomes [12].

Rehabilitation generally encompasses a comprehensive care approach aimed at reducing disability, enhancing participation in activities of daily living, improving functional abilities, preventing further functional deterioration, and maximizing independence [13, 14]. Physical therapy within rehabilitation includes positioning, supportive devices, and prescribed exercises, whereas additional treatment modalities for pain and spasticity may involve pharmacotherapy, botulinum toxin injections, nerve blocks, electrical stimulation, splints, serial casting, and surgical interventions [15–17]. Nevertheless, a significant proportion of stroke survivors will continue to experience some degree of neurological impairment 5 years post‐stroke [18, 19], indicating that novel complementary methods may be necessary to enhance patient outcomes following a stroke.

In recent years, the clinical application of exoskeleton lower limb robots has garnered increasing attention. These robotic devices deliver consistent rehabilitation support to patients through predefined parameters, mitigating issues such as variability in therapists′ skill levels and fatigue. In comparison to conventional rehabilitation methods, robotic interventions provide a standardized rehabilitation regimen [20]. Functional rehabilitation‐oriented lower limb exoskeleton robots can play a significant role in facilitating weight relief and promoting standardized physiological gait patterns during rehabilitation, thereby enabling patients to progressively regain lower limb motor function and attain autonomous walking following neurological injury [21, 22]. The LiteStepper unilateral‐lower limb exoskeleton rehabilitation training robot represents an innovative device for gait rehabilitation. Its primary operational principle involves the unaffected limb being equipped with a lightweight, imperceptible intelligent sensing system while the affected limb is supported by a powered unit. By integrating intelligent gait learning algorithms, patient motion intention recognition technology, multisensor fusion technology, and lightweight wearable exoskeleton fabrication techniques, the device enhances user experience and engagement in rehabilitation based on motor relearning theories. The system promotes the patient′s active learning capacity, improving balance and restoring normal gait through the interaction between the unaffected and affected limbs, as well as human‐machine integration [23, 24]. However, the exact clinical evidence supporting that the unilateral‐lower limb exoskeleton rehabilitation training robot is superior to conventional rehabilitation is still unclear, and further research is needed to verify this. Therefore, this study is aimed at evaluating the effects of a unilateral‐lower‐limb exoskeleton rehabilitation training robot on balance and lower limb function after stroke.

## 2. Material and Methods

### 2.1. Study Design and Participants

This pilot study included stroke patients with hemiplegia who were hospitalized at Tongji University Yangzhi Rehabilitation Hospital between October 2022 and December 2023 to participate in this pilot study. The study was approved by the Ethics Committee of Tongji University Yangzhi Rehabilitation Hospital (Approval No. #EC Form045). All participants provided written informed consent before being included in the study.

Stroke was diagnosed based on neurological examination, brain imaging (such as computed tomography [CT] or magnetic resonance imaging [MRI]). For ischemic stroke, evidence of acute cerebral infarction was required on imaging, and for hemorrhagic stroke, the presence of intracerebral hemorrhage was confirmed. Eligible stroke patients were informed about the trial. Inclusion criteria were as follows: (1) confirmed diagnosis of stroke with hemiplegic (ischemic or hemorrhagic) 3–12 months postacute onset; (2) ability to understand study procedures and provide informed consent; (3) age between 18 and 75 years; (4) stable vital signs and overall health status, capable of tolerating low‐intensity sit‐and‐stand training; (5) weight not exceeding 100 kg and height between 1.50 and 1.90 m; (6) ability to use a lower limb rehabilitation training robot with assistance; and (7) no significant limitations in passive range of motion for the hip, knee, and ankle joints, with the ankle in a neutral position during passive assessment.

Exclusion criteria included (1) severe joint mobility limitations impeding walking; (2) skin damage or infection at contact points with the rehabilitation robot; (3) severe internal medical conditions; (4) significant cognitive impairment interfering with training cooperation; (5) other contraindications or complications adversely affecting gait training; (6) pregnancy, planning pregnancy, or breastfeeding; (7) participation in another clinical trial within the month prior to enrollment; and (8) other conditions deemed inappropriate.

### 2.2. Interventions

Patients were randomly assigned to either the control group or the group receiving combined unilateral‐lower limb exoskeleton rehabilitation training robot‐assisted traditional rehabilitation (robot group), with 18 patients in each group. The control group′s training plan was developed based on the patient′s rehabilitation assessment results to select appropriate rehabilitation measures. The training content includes active movement, weight bearing on the affected limb, center of gravity transfer, balance function, knee joint control, and dorsiflexion strength training. Active movement involves flexion and extension exercises for the hip, knee, and ankle joints, with each set of exercises repeated 10–15 times, completing three sets. Weight bearing on the affected limb begins partially, gradually increasing the duration and proportion. Center of gravity transfer training is conducted between parallel bars, with each set repeated 10 to 15 times for a total of three sets. Balance training includes both static and dynamic components, with dynamic training involving obstacle crossing for approximately 20 min. Knee joint exercises use resistance bands to enhance muscle strength, with each set repeated 10–15 times for a total of three sets. Dorsiflexion strength training involves resistance bands and tiptoe standing to improve walking ability, with each set repeated 10–15 times for a total of three sets. These training exercises is aimed at comprehensively enhancing the patients′ motor functions.

**Figure 1:**
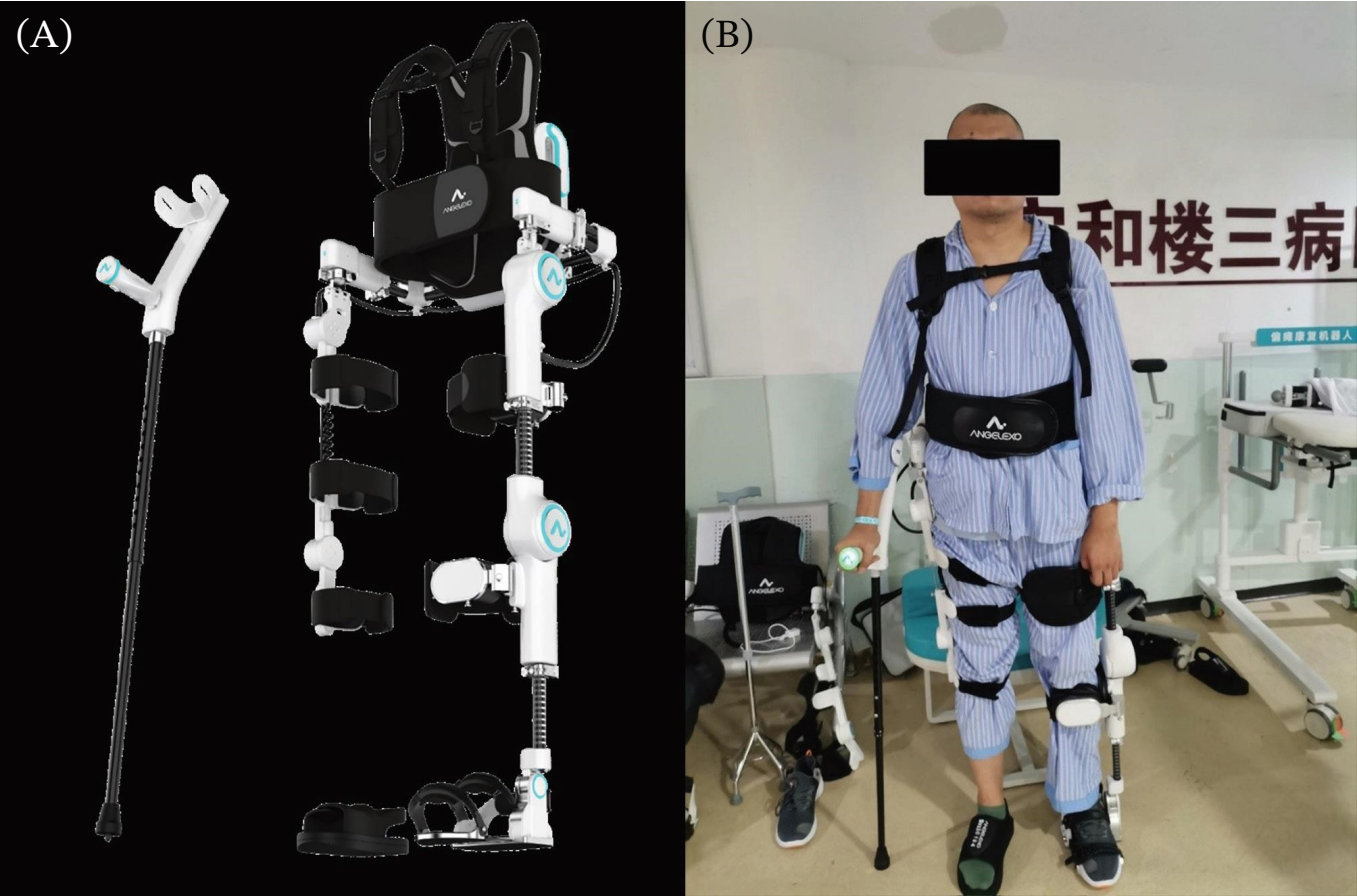
Schematic diagram of the unilateral‐lower limb exoskeleton rehabilitation training robot. (A). Unilateral‐lower limb exoskeleton rehabilitation training device. (B). Patient undergoing rehabilitation training with the unilateral‐lower limb exoskeleton device, assisted by a therapist.

Patients in the robot group received unilateral‐lower limb exoskeleton rehabilitation training robot training (LiteStepper; Zhengzhou Angelexo Scientific Co. Ltd., China) in addition to traditional rehabilitation training, under the guidance of professional therapists [25, 26]. The robot training sessions lasted 20 min each, once daily, for a total of 20 sessions. The unilateral‐lower limb exoskeleton rehabilitation robot‐assisted training begins with a warm‐up phase, during which the patient is assisted by a therapist to wear and adjust the exoskeleton device to ensure comfort and safety (Figure 1). During the training, the robot is programed to assist the patient in performing lower limb movement patterns, including hip flexion and extension, knee joint bending and straightening, and ankle dorsiflexion and plantarflexion. The speed and range of motion are adjusted according to the patient′s recovery level, adhering to the principles of progressive training while providing appropriate resistance during knee joint training to enhance muscle strength. The exoskeleton robot precisely controls the motion angles and forces of the ankle joint, providing targeted training for patients with varying degrees of injury. Through real‐time monitoring and data collection, therapists can promptly adjust training parameters to ensure the effectiveness and safety of the training. This real‐time monitoring and adjustment are key to optimizing exoskeleton‐assisted rehabilitation training [27].

### 2.3. Outcome Measurements

Evaluations were conducted prior to treatment and again 4 weeks posttreatment. The primary outcome measure was the Berg balance scale (BBS) scores. The secondary outcome measures included the time taken to complete a 10‐m walk, the distance covered in 6 min, parameters derived from three‐dimensional gait analysis, the psychological states of the patients, and reported levels of pain. Furthermore, any adverse reactions or complications observed during the treatment process were systematically documented. The evaluators involved in this study did not participate in the treatment process for the patients, and the data analysts were blinded to the group assignments to mitigate potential bias.

The BBS comprises 14 items, each evaluated on a scoring range from 0 to 4, yielding a maximum achievable score of 56 points. Elevated scores are indicative of superior balance function [28]. The three‐dimensional gait analysis encompassed measurements of hip flexion angle, knee flexion angle, ankle dorsiflexion angle, and pelvic tilt angle [29]. Psychological states were assessed utilizing the Hospital Anxiety and Depression Scale (HADS) [30]. The HADS, developed by Zigmond and Snaith in 1983 [31], measures depression and anxiety in hospitalized patients through 14 self‐reported items (seven pertaining to anxiety and seven to depression) rated on a 4‐point Likert scale (ranging from 0 to 3). This results in a scoring system for each subscale that spans from 0 to 21. The presence of anxiety and depression is categorized as absent, mild, moderate, or severe, corresponding to scores of 0–7, 8–10, 11–14, and 15–21, respectively [32]. Additionally, pain levels were evaluated using the visual analogue scale (VAS) [33]. The VAS consists of a 10‐cm line marked at each end with values of 0 and 10, along which the patient indicates the intensity of pain experienced. A ruler is employed to measure the distance from the 0 mark, thereby determining the pain score [34]. Pain is classified as absent, mild, or moderate to severe for scores of 0–0.9, 1.0–3.9, and 4.0–10.0 cm, respectively [35]. All assessments were conducted by the same therapist both prior to and following treatment to evaluate changes in lower limb motor function, balance function, and walking ability.

### 2.4. Sample Size

The sample size was calculated using professional statistical methods. For the comparison of continuous variables between the two groups in this study, a two‐independent‐sample approach was adopted. Based on pilot study data and clinical experience, the standard deviation (SD) of the primary measure variables was estimated to be around 2, and the minimum clinically meaningful difference between the two groups was set at 2. With a two‐sided significance level of *α* = 0.05 (corresponding to *Z*1 − *α*/2 = 1.96) and a power of 80% (1 − *β* = 0.8, corresponding to *Z*1 − *β* = 0.84), the initial calculation suggested that approximately 16 subjects were needed per group. However, considering a potential dropout rate of 10% during the study, adjustments were made. After accounting for this dropout rate, it was determined that 18 patients should be included in each group. In total, 36 patients were recruited for this study. This sample size was selected to ensure that the study had sufficient statistical power to detect significant differences between the two treatment groups.

### 2.5. Statistical Analysis

Statistical analysis was conducted using SPSS Version 22.0 (IBM Corp., Armonk, New York, United States). Continuous variables that followed a normal distribution and had homogeneity of variance were expressed as mean ± SD and analyzed using the *t*‐test. Nonnormally distributed continuous variables were expressed as median (interquartile range) and analyzed using the nonparametric rank‐sum test. Categorical variables were expressed as *n* (%), and group comparisons were conducted using the *χ* ^2^ test. A two‐sided *p* value of less than 0.05 was considered statistically significant. Two‐way repeated‐measures analysis of variance (ANOVA) was used to examine the Group × Time interaction effect of outcome measures, followed by paired *t*‐test for within‐group comparisons and independent samples *t*‐test for between‐group comparison of improvement amplitude.

## 3. Results

### 3.1. Characteristics of the Patients

**Figure 2:**
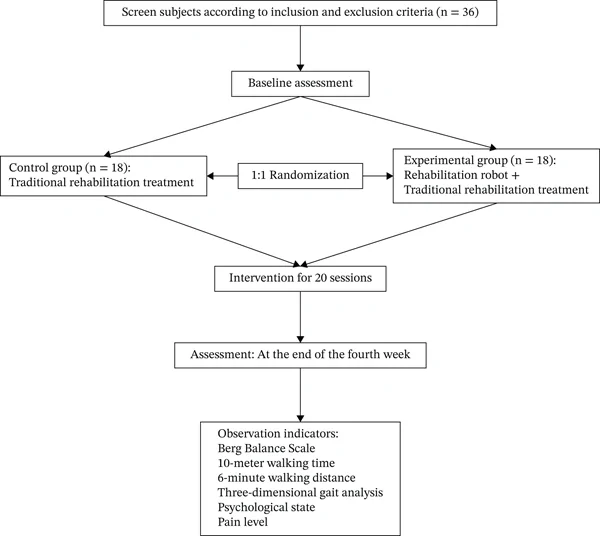
Flowchart of the patient selection and allocation process in the study.

A total of 36 stroke patients with hemiplegic in the recovery phase were included in the study, comprising 25 males and 11 females. Following the categorization based on their selected rehabilitation method, each group consisted of 18 patients. The detailed patient selection and allocation process was shown in Figure 2. Statistical analysis indicated no significant differences between the two groups regarding demographic characteristics (gender, age, duration of illness, or lesion location) and baseline functional outcome measures (BBS, gait parameters, HADS scores, VAS pain score) (Table 1). Stroke lesion topography was recorded and classified into basal ganglia lesions and nonbasal ganglia lesions. The nonbasal ganglia category included cortical lesions, subcortical white matter lesions and brainstem lesions. Given the limited sample size, further subdivision was not performed.

**Table 1:** Baseline characteristics of patients in the two groups.

Variable	Control (*n* = 18)	Robot (*n* = 18)	*p*
Demographic characteristics			
Sex			
Male	12 (66.7%)	13 (72.2%)	0.717
Female	6 (33.3%)	5 (27.8%)	
Age (years)	56.60 ± 13.20	53.50 ± 12.90	0.481
Disease duration (months)	7.33 ± 3.19	7.40 ± 3.21	0.948
Lesion location			
Basal ganglia region	10 (55.6%)	12 (66.7%)	0.494
Nonbasal ganglia region (cortex, subcortical white matter, brainstem)	8 (44.4%)	6 (33.3%)	
Functional outcome measures at baseline			
Berg score	36.78 ± 4.91	38.94 ± 5.96	0.244
6‐min walking distance (m)	112.33 ± 17.96	115.78 ± 19.14	0.581
10‐m walking time (s)	34.08 ± 9.89	32.99 ± 10.10	0.746
Hip flexion angle (°)	6.51 ± 2.30	6.36 ± 3.33	0.876
Knee flexion angle (°)	15.46 ± 6.95	15.58 ± 7.52	0.961
Pelvic tilt angle (°)	4.26 ± 2.33	4.13 ± 2.34	0.868
Ankle dorsiflexion angle (°)	30.76 ± 9.04	31.28 ± 8.59	0.861
HADS/A	8.67 ± 2.22	8.72 ± 2.30	0.948
HADS/D	14.56 ± 2.36	14.94 ± 2.56	0.646
VAS	3.61 ± 1.20	3.83 ± 1.25	0.594

*Note:* Chi‐square test for categorical variables, independent samples *t*‐test for continuous variables.

Abbreviations: HADS/A, Hospital Anxiety and Depression Scale, anxiety; HADS/D, Hospital Anxiety and Depression Scale, depression; VAS, visual analogue scale.

Two‐way repeated‐measures ANOVA revealed significant Group × Time interaction effects for BBS, 6‐min walking distance, 10‐m walking time, hip flexion angle, knee flexion angle, ankle dorsiflexion angle, and HADS‐D score (all p < 0.05), whereas no significant interaction was observed for HADS‐A and VAS scores (p > 0.05) (Table 2). After 4 weeks of treatment combining unilateral‐lower limb exoskeleton rehabilitation robot with conventional therapy, patients demonstrated significant improvements in balance function, increasing from 38.94 ± 5.96 to 44.44 ± 5.48 (p = 0.007); 6‐minute walking distance, extending from 115.78 ± 19.14 m to 131.67 ± 25.11 m (p = 0.040); and walking time, decreasing from 32.99 ± 10.10 s to 24.17 ± 9.96 s (p = 0.013). Joint mobility also improved significantly, with hip flexion angle increasing from 6.36°±3.33° to 9.93°±4.87° (p < 0.001); knee joint angle from 15.58°±7.52° to 20.86°±8.69° (p < 0.001); pelvic mobility from 4.13°±2.34° to 6.71°±2.73° (p < 0.001); and ankle joint angle changing from 31.28°±8.59° to 26.46°±10.25° (p = 0.002). Additionally, depressive symptoms were significantly reduced (p < 0.001). However, no significant changes were observed in anxiety and pain scores (p > 0.05) (Table 3). In contrast, patients receiving conventional therapy alone showed improvements only in walking time (from 34.08 ± 9.89 s to 27.25 ± 9.11 s, p = 0.040), knee joint angle (from 15.46°±6.95° to 18.09°±7.46°, p = 0.036), ankle joint mobility (from 30.76°±9.04° to 28.85°± 6.21°, p = 0.002), and depressive symptoms (p > 0.05), with no significant changes in balance function (Table 4).

**Table 2:** Group × Time interaction effects of two‐way repeated‐measures ANOVA for all outcome measures.

Outcomes	Degree of freedom	F	*p*
Berg score	1, 34	15.54	< 0.001
6‐min walking distance (m)	1, 34	6.28	0.017
10‐m walking time (s)	1, 34	5.23	0.030
Hip flexion angle (°)	1, 34	6.21	0.018
Knee flexion angle (°)	1, 34	4.28	0.046
Pelvic tilt angle (°)	1, 34	2.05	0.161
Ankle dorsiflexion angle (°)	1, 34	5.13	0.030
HADS/A	1, 34	0.02	0.893
HADS/D	1, 34	6.57	0.015
VAS	1, 34	0.02	0.894

*Note:* All effects represent Group × Time interaction; df = 1, 34 (between‐group df = 1, error df = 34); *p* < 0.05 was considered statistically significant.

Abbreviations: HADS/A, Hospital Anxiety and Depression Scale, anxiety; HADS/D, Hospital Anxiety and Depression Scale, depression; VAS, visual analogue scale.

**Table 3:** Comparison of the outcomes before and after treatment in the robot group.

Outcomes	Robot rehabilitation (*n* = 18)		*t*	*p*
	Before	After		
Berg score	38.94 ± 5.96	44.44 ± 5.48	2.882	0.007
6‐min walking distance (m)	115.78 ± 19.14	131.67 ± 25.11	2.135	0.040
10‐m walking time (s)	32.99 ± 10.10	24.17 ± 9.96	2.638	0.013
Hip flexion angle (°)	6.36 ± 3.33	9.93 ± 4.87	6.581	< 0.001
Knee flexion angle (°)	15.58 ± 7.52	20.86 ± 8.69	4.577	< 0.001
Pelvic tilt angle (°)	4.13 ± 2.34	6.71 ± 2.73	6.615	< 0.001
Ankle dorsiflexion angle (°)	31.28 ± 8.59	26.46 ± 10.25	3.665	0.002
HADS/A	8.72 ± 2.30	8.28 ± 1.57	1.512	0.149
HADS/D	14.94 ± 2.56	12.06 ± 2.24	7.023	< 0.001
VAS	3.83 ± 1.25	3.67 ± 0.77	0.511	0.616

*Note:* *p* < 0.05 was considered statistically significant.

Abbreviations: HADS/A, Hospital Anxiety and Depression Scale, anxiety; HADS/D, Hospital Anxiety and Depression Scale, depression; VAS, visual analogue scale.

**Table 4:** Comparison of the outcomes before and after treatment in the traditional rehabilitation group.

Outcomes	Traditional rehabilitation (n = 18)		*t*	*P*
	Before	After		
Berg score	36.78 ± 4.91	39.89 ± 4.85	1.912	0.064
6‐min walking distance (m)	112.33 ± 17.96	122.06 ± 22.30	1.442	0.159
10‐m walking time (s)	34.08 ± 9.89	27.25 ± 9.11	2.157	0.040
Hip flexion angle (°)	6.51 ± 2.30	8.23 ± 2.83	1.507	0.229
Knee flexion angle (°)	15.46 ± 6.95	18.09 ± 7.46	3.627	0.036
Pelvic tilt angle (°)	4.26 ± 2.33	5.70 ± 3.54	3.058	0.055
Ankle dorsiflexion angle (°)	30.76 ± 9.04	28.85 ± 6.21	11.180	0.002
HADS/A	8.67 ± 2.22	8.17 ± 1.82	1.767	0.095
HADS/D	14.56 ± 2.36	12.94 ± 2.26	5.720	< 0.001
VAS	3.61 ± 1.20	3.50 ± 0.86	0.437	0.668

*Note:* *p* < 0.05 was considered statistically significant.

Abbreviations: HADS/A, Hospital Anxiety and Depression Scale, anxiety; HADS/D, Hospital Anxiety and Depression Scale, depression; VAS, visual analogue scale.

The difference in BBS score changes between groups (traditional group 3.11 ± 1.50 vs. robot group 5.50 ± 2.09) was statistically significant (p < 0.001), indicating more substantial benefits for patients receiving robot‐assisted conventional rehabilitation therapy. Compared with the control group, the robot group demonstrated greater improvements in 6‐min walking test distance change (9.73 ± 5.83 m vs. 15.89 ± 8.64 m, p = 0.017), 10‐m walking time reduction (6.83 ± 1.81 s vs. 8.82 ± 3.22 s, p = 0.030), and all gait parameters, including hip flexion angle (1.72°±2.15° vs. 3.57°±2.30°, p = 0.018), knee flexion angle (2.63°±2.37° vs. 5.28°±4.89°, p = 0.046), and ankle dorsiflexion angle (1.09°±4.19° vs. 4.81°±5.57°, p = 0.030). Furthermore, the robot group exhibited significantly greater improvement in depressive symptoms compared with the traditional group (1.61 ± 1.20 vs. 2.89 ± 1.75, p = 0.015). However, differences in anxiety and pain VAS score changes between the two groups were not statistically significant (all p > 0.05) (Table 5).

**Table 5:** Comparison of improvement amplitude of the outcomes between the two groups.

Outcomes	Changes after treatment		*t*	*p*
	Traditional (*n* = 18)	Robot (*n* = 18)		
Berg score	3.11 ± 1.50	5.50 ± 2.09	3.942	< 0.001
6‐min walking distance (m)	9.73 ± 5.83	15.89 ± 8.64	2.507	0.017
10‐m walking time (s)	6.83 ± 1.81	8.82 ± 3.22	2.286	0.030
Hip flexion angle (°)	1.72 ± 2.15	3.57 ± 2.30	2.493	0.018
Knee flexion angle (°)	2.63 ± 2.37	5.28 ± 4.89	2.069	0.046
Pelvic tilt angle (°)	1.44 ± 2.94	2.58 ± 1.66	1.433	0.161
Ankle dorsiflexion angle (°)	1.09 ± 4.19	4.81 ± 5.57	2.264	0.030
HADS/A	0.50 ± 1.20	0.44 ± 1.25	0.136	0.893
HADS/D	1.61 ± 1.20	2.89 ± 1.75	2.563	0.015
VAS	0.11 ± 1.08	0.17 ± 1.38	0.134	0.894

*Note:* *p* < 0.05 was considered statistically significant.

Abbreviations: HADS/A, Hospital Anxiety and Depression Scale, anxiety; HADS/D, Hospital Anxiety and Depression Scale, depression; VAS, visual analogue scale.

## 4. Discussion

The results indicated that the use of a unilateral‐lower limb exoskeleton rehabilitation training robot, combined with traditional rehabilitation therapy, significantly enhanced the lower limb motor function in stroke patients compared with traditional rehabilitation therapy alone. Specifically, the use of the robot provided additional benefits in joint range of motion (hip, knee, and ankle), balance function, walking speed, and walking distance of the affected limb, while also significantly improving depressive symptoms.

Research conducted by Jin et al. indicates that robot‐assisted traditional rehabilitation therapy surpasses conventional rehabilitation in terms of the BBS, Functional Ambulation Classification (FAC) scale, 6‐min walk test, and Barthel index [23]. Similarly, another trial focusing on subacute stroke patients demonstrated that machine‐assisted rehabilitation yielded superior improvements in BBS, FAC, 6‐min walk test, lower limb Fugl‐Meyer assessment, and modified Barthel index when compared with traditional rehabilitation methods [24]. This study reaffirmed these findings and further investigated the specific benefits for patients receiving robot‐assisted traditional rehabilitation therapy, particularly regarding the joint range of motion in the affected limb. Such improvements may be attributed to the high repeatability, efficiency, and consistency of the unilateral‐lower limb exoskeleton rehabilitation robot [20]. During training, this technology provides strength support, facilitating precise and repetitive walking task training. Personalized exercise programs guide the rehabilitation process, thereby promoting the restoration of normal gait. Additionally, the exoskeleton robot can objectively and continuously monitor patient metrics and progress throughout the rehabilitation process, offering active, assistive, and passive movement modes through a real‐time feedback system to maximize improvements in muscle motor function and coordination in the affected limb [36].

In contrast, traditional rehabilitation training involves therapists manually controlling the patient’s pelvis, hip, knee, and ankle joints to simulate natural repetitive walking. This method imposes certain restrictions on the physiological movement of the pelvis and trunk, making it challenging to organically integrate the three essential elements of walking: weight bearing, balance, and stepping [37]. Furthermore, manual joint control results in a lack of systematic internal and external sensory feedback, leading to actions that are neither natural nor coherent. Consequently, although traditional rehabilitation training can enhance lower limb function in stroke patients, its therapeutic outcomes are often suboptimal. This method also necessitates significant physical effort from therapists and fails to engage patients sufficiently, resulting in a lack of interest and motivation. Although improvements in gait and mobility are noted as training duration increases, high‐intensity and repetitive training can eventually become demanding and may lead to abnormal gait patterns [38, 39]. Therefore, in this study, although traditional rehabilitation therapy improved the 10‐m walking time and knee and ankle flexion angles, it had no significant impact on BBS, the distance of the 6‐min walk test, hip flexion angle, or pelvic tilt angle.

Additionally, it is noteworthy that this study assessed the psychological state of patients. Compared with traditional rehabilitation therapy, the robot‐assisted rehabilitation group demonstrated significantly greater improvement in depressive symptoms following treatment. After experiencing illness, patients frequently encounter limitations in physical health and activity levels, which can result in increased dependency, exacerbated depression, and diminished quality of life. The integration of robotic and traditional rehabilitation therapies offers substantial support by compensating for diminished intrinsic functions, thereby facilitating enhancements in patients′ psychological well‐being [40]. This finding suggests that future rehabilitation strategies should prioritize the incorporation of exoskeletons and the development of tailored rehabilitation programs to enhance both the physical and mental well‐being of patients.

Furthermore, the study carries several limitations. Only short‐term rehabilitation outcomes were assessed, and long‐term effects remain unknown. In addition, we primarily focused on lower limb motor recovery without systematic evaluation of sensory function, interlimb coordination and other domains. The relatively small sample and narrow participant spectrum may limit the generalizability of our findings. As an observational investigation, treatment selection depended on participant preference; accordingly, disparities in socioeconomic background or understanding of robotic rehabilitation could confound clinical outcomes. Clinically, lacunar infarction frequently produces mild deficits without overt hemiplegia, and for this reason, unilateral‐lower limb exoskeleton training yields limited benefits for such patients. We thus enrolled only participants with hemiplegia secondary to nonlacunar ischemic stroke and excluded those with pure motor lacunar stroke. Whether rehabilitation trajectories differ between lacunar and nonlacunar groups merits further scrutiny, given that lacunar lesions typically predict superior motor recovery [41]. When feasible, future trials may include eligible lacunar stroke patients with hemiplegia for comparative analysis.

Another notable constraint concerns the age spectrum of recruited patients. Participant entry was restricted to ages 18–75 years, whereas no adults aged ≥ 85 years were enrolled. Adults older than 85 years have diminished physical reserve, elevated fall risk and reduced motor plasticity [42], which means protocols optimized for younger adults cannot be directly transferred to this population. Therefore, developing customized low‐intensity, lightweight exoskeleton regimens tailored for the oldest‐old stroke survivors warrants dedicated research.

Taken together, large‐scale, multicenter randomized controlled trials covering varied stroke subtypes and severity grades are required. Extended follow‐up and multidimensional outcome assessment will strengthen the overall evidence base. Upcoming work can incorporate longitudinal monitoring, multimodal neuroimaging such as fMRI and DTI to unpack underlying mechanisms of neural plasticity, alongside adaptive training algorithms modulated according to individual motor performance and fatigue. Beyond that, trials may also test combinatorial therapeutic approaches to identify potential synergistic effects when exoskeleton training is paired with neuromuscular electrical stimulation, mirror therapy or cognitive rehabilitation. Collectively, such efforts will validate our results and define the broader clinical scope for robotic‐assisted rehabilitation.

## 5. Conclusion

In conclusion, this study provides evidence that the integration of a unilateral‐lower limb exoskeleton rehabilitation robot with traditional rehabilitation training facilitates a more effective recovery of lower limb motor function in stroke patients with hemiplegia compared with traditional rehabilitation training alone. Furthermore, the findings suggest that future rehabilitation strategies should incorporate exoskeleton technologies and emphasize the development of individualized rehabilitation programs to enhance both the physical and mental well‐being of patients.

## Author Contributions

Shaoting Zhou, Sen Wang, and Xueying Qu carried out the studies and participated in collecting data. Shaoting Zhou and Jing Fan performed the statistical analysis and drafted the manuscript. Hanlin Xu and Linyu Chen contributed to the assessment of patient rehabilitation. Lingyu Liu contributed to manuscript editing, conceptualization, and supervision. Shaoting Zhou and Sen Wang contributed equally to this work.

## Funding

This study was supported by the National clinical key specialty construction project of China (Z155080000004); Songjiang District Science and Technology Commission Project of Shanghai (2023SIKIGG105); Sunshine Clinical Research Cultivation Project of Yangzhi Rehabilitation Hospital Affiliated to Tongji University (2024CRTS003); and Shanghai Municipal Health Commission (10.13039/100017950, SHKFJS002A).

## Disclosure

All authors read and approved the final manuscript.

## Ethics Statement

The study was approved by the Ethics Committee of Tongji University Yangzhi Rehabilitation Hospital (Approval No. #EC Form045). All participants provided written informed consent before being included in the study. I confirm that all methods were performed in accordance with the relevant guidelines. All procedures were performed in accordance with the ethical standards laid down in the 1964 Declaration of Helsinki and its later amendments.

## Consent

The authors have nothing to report.

## Conflicts of Interest

The authors declare no conflicts of interest.

## Data Availability

All data generated or analyzed during this study are included in this published article.
